# Emerging Pattern of Post-COVID-19 Parosmia and Its Effect on Food Perception

**DOI:** 10.3390/foods11070967

**Published:** 2022-03-27

**Authors:** Jane K. Parker, Lisa Methven, Robert Pellegrino, Barry C. Smith, Simon Gane, Christine E. Kelly

**Affiliations:** 1 Department of Food and Nutritional Sciences, University of Reading, Reading RG6 6DZ, UK; l.methven@reading.ac.uk (L.M.); chris@abscent.org (C.E.K.); 2 Monell Chemical Senses Center, Philadelphia, PA 19104, USA; rpellegrino@monell.org; 3 Centre for the Study of the Senses, Institute of Philosophy, School of Advanced Study, University of London, London WC1E 7HU, UK; barry.smith@sas.ac.uk; 4 Royal National Ear, Nose and Throat and Eastman Dental Hospitals, University College London Hospital, 47-49 Huntley St., London WC1E 6DG, UK; simongane@nhs.net; 5 AbScent, 14 London Road, Andover SP10 2PA, UK

**Keywords:** COVID-19, olfactory distortions, parosmia, trigger foods, disgust, valence

## Abstract

Olfactory dysfunction is amongst the many symptoms of Long COVID. Whilst most people that experience smell loss post COVID-19 recover their sense of smell and taste within a few weeks, about 10% of cases experience long-term problems, and their smell recovery journey often begins a few months later when everyday items start to smell distorted. This is known as parosmia. The aim of this study was to identify the key food triggers of parosmic distortions and investigate the relationship between distortion and disgust in order to establish the impact of parosmia on diet and quality of life. In this cross-sectional study (*n* = 727), respondents experiencing smell distortions completed a questionnaire covering aspects of smell loss, parosmia and the associated change in valence of everyday items. There was a significant correlation between strength and disgust (*p* < 0.0001), and when the selected items were reported as distorted, they were described as either unpleasant or gag-inducing 84% of the time. This change in valence associated with loss of expected pleasure and the presence of strange tastes and burning sensations must certainly lead to changes in eating behaviours and serious longer-term consequences for mental health and quality of life.

## 1. Introduction

Sense of smell guides our selection and appreciation of food and plays a dominant role in flavour perception [1]. When that sense is missing or impaired, the consequences are far-reaching. The impact of olfactory dysfunction on diet, quality of life, and interpersonal relationships is well-documented [2]; never more so as witnessed poignantly during the COVID-19 pandemic, with olfactory loss recognised worldwide as one of the official symptoms of COVID-19. Burges Watson et al. identified from a co-created study, based on social media posts of those with post-COVID-19 alterations in taste and smell, three broad concerns: (i) a radically altered experience of food and eating, (ii) difficulty in making sense of the altered experience, and (iii) altered relationships to the world [3]. They concluded that in cases where the sense of smell is not recovered within 2–3 weeks, the effect is not mild, given that it may last for months, and it has “serious implications for food, eating, health, work and well-being”.

Prior to the COVID-19 pandemic, olfactory disorders were a largely unrecognised problem, even though they prevailed in up to 23% of the population [4]. Such disorders are a known consequence of viral illness or infection [5] and are often referred to as post-infectious or post-viral olfactory dysfunction. In long-term cases (experienced by about 10% of all COVID-19 cases), there is an initial loss of sense of smell (anosmia), and as the recovery process begins, typically 2–3 months after the initial loss [6], many experience qualitative olfactory disorders. Parosmia is one such qualitative disorder which alters an individual’s perception of odours in such a way that every day smells are commonly described as “distorted”. These distortions are often associated with strong dislike or disgust and can persist in some cases for up to 10 years [7]. In extreme cases of parosmia, some triggers can provoke nausea and vomiting [8]. However, it is generally recognised as a sign of recovery and has been identified recently as an independent predictor for complete recovery [9].

We present here the impact of this condition on the perception of food within the greater context of the onset, symptoms, and duration of COVID-19-related olfactory disorders. The main aim of this investigation is to examine the late-emerging pattern of qualitative olfactory dysfunction and its effect on the perception of common foods and beverages. Our focus in this paper is on parosmia, the foods that trigger the distortions associated with parosmia and, in particular, on trying to understand the relationships between distortion and disgust. We explore differences between cases which are post-COVID-19 and cases which were attributed to other viral infections, and investigate characteristics of parosmia. An explanation of the aetiology of the disease is important in understanding the impact on food; hence, the first part of this paper will address these biomedical issues.

## 2. Materials and Methods

### 2.1. Ethics and Recruitment

All subjects gave their informed consent for inclusion before they participated in the study. The investigations were carried out following the rules of the Declaration of Helsinki of 1975, and the protocol was approved by the School of Chemistry, Food and Pharmacy Research ethics committee of the University of Reading on 10 June 2020 (study number 29.2020). It was registered under the US Library of Medicine as trial NCT04868435.

This is a cross-sectional study. Participants were recruited through ENT clinics, Facebook (AbScent Parosmia and Phantosmia Support group and personal accounts), and Twitter between 19 June 2020 and 5 September 2021. Volunteers aged 18 or over who were experiencing smell distortions or for whom everyday things smelled different, odd, or disgusting were invited to participate in the fully anonymised survey. Entry into the study was dependent on the participants completing a standard unlinked online consent form which then took them to the survey landing page. Participation in this online study was voluntary, and respondents received no remuneration. The survey was carried out on Compusense (West Guelph, ON, Canada).

### 2.2. The Questionnaire

After completing the consent form, respondents completed the six-part survey. Demographic data (age, gender, country of residence, ethnic group, and smoking status) were collected in Section 1, whilst Section 2 asked questions about the speed, timings, and aetiology of the respondents’ initial loss of smell (anosmia). They were asked when they lost their sense of smell (date) and the likely aetiology of their symptoms (COVID-19, other viral illness, accident (including head or brain injury), unexplained (or idiopathic), or other/do not know). If the cause was COVID-19 or another viral infection, the speed of loss was reported as one of 4 categories: very suddenly before the onset of other symptoms of infection, very suddenly during infection, very suddenly after infection, or gradually. In cases where it was attributed to COVID-19, further questions were asked about diagnosis (PCR, antibody test, or no test) and severity. Section 3 asked about the onset of parosmia, whether it had been preceded by any partial or full recovery of the sense of smell (none, a few hints, any partial or full recovery), and whether the symptoms fluctuated significantly (four categories: no fluctuation, infrequent or minor fluctuations, significant daily random fluctuations, significant but generally get better during the day). In Section 4, respondents were asked to indicate whether they could taste salt and sugar (three categories: taste as normal, taste weaker, or cannot taste), whether they could detect heat in spices (yes/no), and whether they had experienced any metallic taste (yes/no) or burning sensations in their nose or throat (yes/no).

The core survey (Section 5) concerned the respondents’ perception of 14 foods (referred to as the 14 “set triggers”) which had been pre-selected based on data from other studies [8,10]. Onion, meat, coffee, and eggs were selected on the basis of being among the most commonly reported trigger foods for parosmia. Chocolate, peanuts, bacon, fried foods, peppers, cucumber, and melon were selected because they were known but less frequent triggers, whereas butter, apple, and rose were selected as examples of “safe” foods and smells that were less likely to trigger parosmia.

For each item, the respondents were asked to record whether they perceived the smell of that item as distorted (four categories: smells like it did before, smells distorted, I cannot smell it at all, or I am not familiar with this food/smell so cannot answer). If either of the last two answers was selected, the survey skipped to the next item. Those who selected “distorted” were asked to provide two to three words to describe the distortion. Next, respondents were asked to rate their hedonic assessment of the smell as pleasant (score = 1), neither pleasant nor unpleasant (2), unpleasant (3), or so bad I want to gag/vomit/leave the room (4). An additional option had been provided in the questionnaire, “This food has always smelt unpleasant”, to allow for those where there was no change in hedonic rating because the item had always been perceived as unpleasant. This option was used in 160 out of 6447 observations, and these data were excluded on the grounds that there had been no change in hedonic valence due to parosmia, but we suspect that this answer may have been misinterpreted by many of the respondents. Lastly, respondents were asked to record the strength of the smell now in comparison to before smell loss: weaker than before (score = 1), same as before (2), or stronger than before (3) with the additional option to report that the intensity fluctuated (not associated with a score).

Faecal odour had been highlighted in a previous publication based on social media [8] as being less unpleasant and more tolerable for some, whilst for others, there was a switch in hedonic valence from repulsive to pleasant. This was explored further by asking about the distortion of faecal odour on a three-category scale (same as before, distorted, or cannot smell) and asking about the hedonic quality on a two-category scale (no longer unpleasant, just as unpleasant as before).

Section 6 of the survey involved a check-all-that-apply (CATA) question covering an additional 20 possible triggers selected to cover a wide range of food, drink and some environmental or personal care items, with the opportunity to add further triggers as free text. The final question gave the respondents the opportunity to add any further comments.

### 2.3. Data Analysis

Data relating to respondents’ demographics, aetiology, onset, and recovery were expressed as total count (*n*) and proportion (%). To investigate associations between the different aetiologies and onset, partial recovery and frequency of fluctuation, contingency tables were prepared on the counts and analysed using Fisher’s Exact test (α = 0.05). To determine whether there were significant differences in taste loss between respondents that had suffered COVID-19 versus other viral infections, the count data were similarly analysed by Fisher’s Exact test (α = 0.05).

The Kruskal–Wallis two-tailed test with multiple pairwise comparisons with a Bonferroni correction was used to determine whether disgust was significantly different between the set triggers. The Kruskal–Wallis two-tailed test with multiple pairwise comparisons using Dunn’s procedure was used to determine whether strength was significantly different between the set triggers (from Section 5). Kruskal–Wallis and Dunn’s procedure were similarly used to determine whether there was a relationship between distortion and both hedonic valence and strength and between strength and hedonic valence. Statistical significance was considered at the 5% level (*p* ≤ 0.05).

Descriptions of distorted food items (including faecal odour) were cleaned, and words were spell-checked with Hunspell using a large English dictionary [11]. For the word clouds, single word or compound adjectives were extracted from the descriptions and qualifiers suggesting qualitative changes (weaker, stronger, faint, etc.) were removed. Obvious synonyms were combined (e.g., gasoline/petrol, garbage/trash/bin, toxic/poisonous, poo/poop/faeces/feces/faecal/fecal odour, synthetic/artificial, cat food/dog food) and words with the same root were combined under one term (e.g., chemical/chemically, earth/earthy, burnt/burning rotten/rotting, but sick and sickly for example were deemed to relate to different smells). The frequencies of the words reported for each trigger were calculated, and words where the frequency per item was never more than 1 were removed. This was carried out for the 14 set triggers as well as for the answers to the question on faecal odour. Words for each were visually represented in word clouds with the size representing the frequency using ggwordcloud [12]. Descriptions of distortions next underwent sentiment analysis using the sentimentR package [13] for each item, were averaged and then compared using ANOVA with post hoc analysis and Tukey’s honest significant difference (HSD).

The 120 words were further split into descriptive words where there was a true description of aroma character (79), hedonic words where there was a clear valence attributed to the word (30), and the remaining words (11) (e.g., indescribable, different, funky, unusual). Principal component analysis (PCA) using covariance was carried out on the frequencies of the descriptive words and on the frequencies of the hedonic words.

Manual counts of the items mentioned in the free text were performed in order to identify the most frequently reported triggers. All those items previously assessed in either the set triggers (14) or the CATA (20) were disregarded, as were complex dishes that contained a number of potential triggers (e.g., curry, falafel, pasta sauce, baked beans), and the focus was on simple ingredients and personal care, home care, or environmental odours. These were counted in a word search, using the word or the root of the word where the word was commonly misspelt or had regional variations. However, each incidence was verified since the contextual significance in which these words were mentioned varied: many people chose to tell us which items were not distorted, or which items came back first, or some words were used as descriptors for others (e.g., coffee smells like bleach, so bleach was disregarded on that occasion).

## 3. Results

### 3.1. Demographic Characteristics

The questionnaire was started by 945 people, 17 preliminary practice runs were removed, as were a further 201 non-completers, leaving 727 respondents who completed the whole survey (78%). All demographic data are shown in Table 1. We note that the demographics are skewed towards white (87%), females (90%), and those living predominantly in the UK (45%) or the USA (41%). These demographics reflect those of the AbScent Facebook groups, which were the major source for recruitment, where the proportion of women responding to the survey reported in [14] was 76%, and 76% were residents in either the UK or the USA.

### 3.2. Origin and Progression of Olfactory Dysfunction

#### 3.2.1. Aetiology

In this study, 92% of the cases of smell loss were attributed to a viral infection which is consistent with data reported in a similar but larger self-selecting cross-sectional study carried out at a similar time showing that for those respondents with parosmia, 89% were post-viral cases [15]. Table 1 shows 83% of the respondents had lost their sense of smell due to COVID-19, whereas only 9% had lost their sense of smell from non-COVID-19 infections. This is not surprising as there has been a surge in cases of post-COVID-19 olfactory dysfunction since the start of the pandemic in January 2020.

#### 3.2.2. Timings of Smell Loss

Most of the respondents (98%) reported the onset of distortions within the past 10 years and 88% within the last 2 years. Most (95%) experienced the onset of parosmia less than 6 months after their initial loss of smell with a mean time of 4.4 months and a median time of 2.8 months, similar to other pre- or post-COVID-19 cases ([16,17], respectively) and consistent with the peak survey response rate in August 2020, just 4 months after the peak in cases of COVID-19 in the UK. The timescale for the remaining 5% spread between 6 months and 24 years.

In all post-viral cases, about half of the respondents (56%) reported their loss of sense of smell as concomitant with other symptoms. However, 21% of post-COVID-19 respondents lost their sense of smell very suddenly preceding onset of other symptoms (Table 2), consistent with data from Borsetto et al. [18], who reported in a systematic review that typically 20% of post-COVID-19 cases experienced a loss of sense of smell as the first symptom. This was rarely the case (2%) for non-COVID-19 post-viral respondents. The use of Fisher’s exact test, which takes into account groups of different sizes, showed this difference was significant at *p* < 0.0001. This early onset was noted by Gane et al. [19], who identified the Isolated Sudden Onset of Anosmia as a novel post-COVID-19 syndrome. Although the difference in the size of the two groups does place some limitations on the conclusions we have drawn, the results are fully in line with other studies. Early onset is the reason why it became so important to recognise loss of sense of smell as an official symptom of COVID-19 to minimise further spread of the disease.

#### 3.2.3. Severity of COVID-19

The majority of post-COVID-19 cases were self-reported as either mild, moderate or asymptomatic, with 19% of respondents reporting severe symptoms and three respondents having been hospitalised (Table 1). The current literature shows that olfactory dysfunction is more prevalent in mild COVID-19, with 86% of patients in a cohort of mild to medium cases reporting olfactory dysfunction [20,21,22], but this is difficult to assess since smell and taste checks were rarely performed on those hospitalised with severe respiratory conditions.

#### 3.2.4. Intermittent Recovery of Olfactory Function

Most of the post-COVID-19 respondents (82%) reported some recovery of their olfactory function prior to the onset of parosmia (Table 3) compared to 27% of non-COVID-19 post-viral respondents. The use of Fisher’s exact test, which takes into account groups of different sizes, showed this difference in pre-parosmia recovery is significantly more prevalent in post-COVID-19 respondents (*p* < 0.0001). However, Borsetto et al. [18] showed the disparity between self-reported olfactory function and objective testing, with examples of significant recovery when objective tests showed little improvement and vice versa. Thus, the terms “full recovery” and “no recovery” need to be treated with caution, but undoubtedly, respondents were acutely aware of changes in their olfactory function. Although these differences in recovery are quite significant, there are limitations in the conclusions drawn arising from the fact that those with non-COVID-19 are not reporting recent changes, leading to a potential memory bias between the two groups.

Furthermore, over half of respondents reported that their symptoms fluctuated, as had been highlighted in the thematic analysis of social media posts [8]. Minor or infrequent fluctuations in symptoms were reported significantly more frequently for post-COVID-19 cases (*p* < 0.001), whereas the lack of recovery and lack of fluctuations were both reported significantly more frequently for non-COVID-19 post-viral cases (both *p* < 0.0001).

Those with hints of recovery were significantly more associated with infrequent minor fluctuations (*p* < 0.0001), and those with partial recovery were significantly more associated with any level of fluctuation than no fluctuation (*p* < 0.0001); thus, the partial recovery is likely to be intermittent, as opposed to a stable increase in olfactory function. This notion of partial but sporadic recovery seems to be more prevalent post-COVID-19 and is characterised by fluctuations in the olfactory dysfunction prior to the full onset of parosmia. Such apparently random changes in olfactory (dys)function remain a puzzle for those who are interested in understanding the underlying mechanisms of parosmia as it is difficult to rationalise with the prevailing theory of widespread destruction and slow (misguided) regeneration of olfactory sensory neurons [16,17,23].

#### 3.2.5. Changes in Taste and Chemesthesis

Parma et al. [24] demonstrated that loss of smell, taste, and trigeminal sensation were all compromised post-COVID-19 with self-reported decreases of 80, 69, and 37%, respectively within 2 weeks of a respiratory illness. However, our data, which, on average, were collected 3 months after smell loss, show very little evidence of (residual) loss of the taste of sugar, salt, and the heat in spices in post-COVID-19 respondents with only 3.5%, 1.3%, and 9.6% of cases, respectively, reporting a loss of these senses. This concurs with previous literature on smell and taste loss post-COVID-19, which shows that post-COVID-19 ageusia improves in most cases after 10 days [25] and taste often improves, whilst olfaction does not [26]. However, taste was weaker for 31% of post-COVID-19 respondents for sweet and 27% for salt. This may be partial recovery or due to the unfamiliarity of experiencing those sensations without the co-presence of smell. The recovery of taste and trigeminal sensation is good news for those struggling to eat, enabling them to take an interest and explore a greater variety in the gustatory and somatosensory properties of foods. However, 45% of all respondents reported the presence of a metallic taste in the mouth, 31% reported burning nasal passages, and 14% reported a burning sensation in the mouth. No significant association was found (*p* > 0.05) for any of these tastes or trigeminal sensations with non-COVID-19 or post-COVID-19 aetiologies. Such distortions of the gustatory and trigeminal senses reinforce the aversion to many foods created by parosmic distortions, further restricting food selection and adversely affecting the eating behaviour and nutritional quality of the diet.

### 3.3. Distortion, Disgust, and Strength of Triggers Foods

#### 3.3.1. Key Trigger Foods

Of the 14 set triggers in the questionnaire, coffee, meat, and onion were the most frequently distorted and least likely to be undetected or normal amongst those who were familiar with the items (Table 4).

#### 3.3.2. Characterisation of Distortions

**Figure A1:**
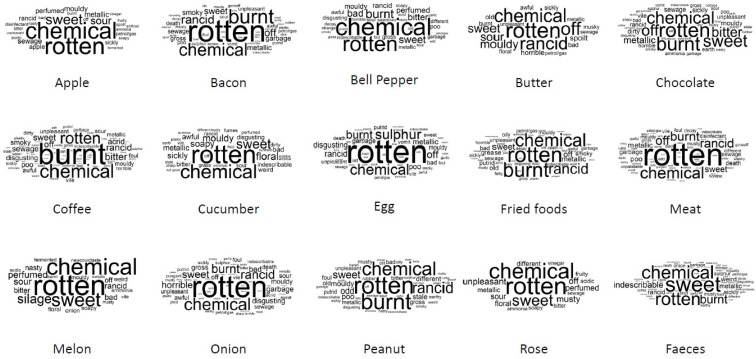
Word clouds representing words used to describe the 14 set triggers and faecal odour. Raw data are provided in Supplementary Table S1.

The three words used most frequently to describe the distortions were “rotten”/”rotting”, “chemical”, and “burnt” used in 14, 11, and 7% of all descriptions. “Burnt” was used more frequently for coffee, whereas “rotten”/”rotting” were used more frequently to describe meat and onions. “Chemical”, which was used across the range of trigger foods, is often a catch-all term discouraged in sensory profiling as, without further definition, it can relate to any number of chemical odours and tends to cover a range of otherwise unidentifiable odours. “Sweet” was the next most frequent (5%) and was the term most frequently reported for faecal odour. The words “indescribable”/”cannot describe” were used 94 times, reflecting the difficulty respondents had in finding appropriate words for smells they consider novel. Indeed, given that most of the support provided for those with parosmia is through online support groups, the selection of some of these descriptors may have been influenced by online discussions. The word frequencies are represented in the word clouds in Figure A1. It is worth noting that many of these terms offered by respondents seem to be chosen for their negative hedonic value (“rotting”) rather than for their a priori resemblance to the actual descriptor. Comments about the indescribability of these parosmic odours support the argument that, often, descriptors used are shorthand for the level of disgust felt. Indeed, previous work has shown that disgust is the highest emotion expressed in descriptions of parosmic triggers [15].

#### 3.3.3. Valence of Distortions

**Figure 1:**
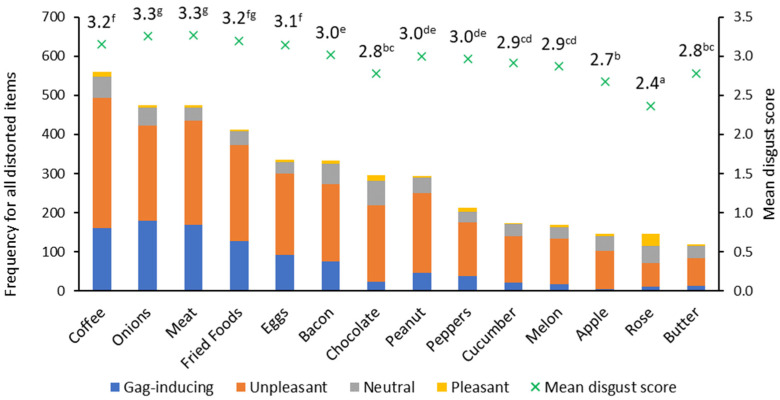
Distortion frequency, hedonic ratings (frequency of counts), and mean disgust score for distorted set triggers. For the mean disgust score, items with the same letters in the superscript are not significantly different from each other using Kruskal–Wallis with Bonferroni correction.

The overall hedonic ratings for all 14 set trigger foods that were described as distorted are shown in Figure 1. In total, 84% of all hedonic responses were unpleasant or gag-inducing, and 16% were rated as pleasant or neutral. Although coffee is most frequently reported as distorted, meat and onions had significantly higher scores for disgust than coffee (*p* = 0.007 and 0.009 respectively). Rose had a significantly lower mean disgust score than any of the other items (*p* ranges from 0.025 (apple) to 0.0001), with about half of those finding it distorted, scoring it as pleasant or neutral.

#### 3.3.4. Strength of Distortions

Coffee, egg, fried food, onion, and meat were rated as significantly stronger than all other triggers except bacon (which was scored separately from meat, originally based on the different molecular composition of its aroma) (*p* < 0.001). Distorted smells were significantly more unpleasant and significantly stronger than non-distorted smells (both *p* < 0.0001). The strongest aromas were significantly less liked (or more disgusting) overall (*p* < 0.0001).

#### 3.3.5. Faecal Odour

Over half of the respondents (60%) reported distortion of faecal odour, whilst 34% reported no smell at all (similar order of magnitude to those who could not smell apple or rose), and only 6% reported no change to faecal odour. Of those that could perceive faecal odour (66%), 30% reported it as not unpleasant anymore.

**Figure 2:**
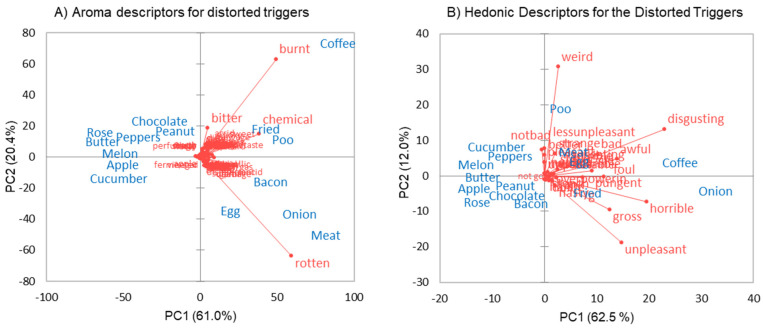
Frequencies of words used to describe the set triggers and faecal odour; PC1 vs. PC2 (**A**) = aroma descriptors, (**B**) = hedonic descriptors. Blue denotes set triggers; red denotes descriptors. Raw data are provided in Supplementary Table S1.

The relationships between the 14 main triggers and faecal odour (denoted as “poo”) were investigated further using PCA. In Figure 2A, the PCA carried out on just the descriptive word frequencies shows a clear separation along PC1 of the common triggers (coffee, fried foods, poo, bacon, meat, onion) from the less common triggers. They are all associated with the descriptor “chemical” (as also shown in the word clouds in Figure A1), but coffee is separated from meat and onions on PC2, being associated with burnt notes, whereas meat and onion are associated with rotting/rotten notes, and fried foods and poo are positioned between the two providing elements of both. In Figure 2B, which shows a PCA based on just the hedonic word frequencies, the separation is quite different with both coffee and onion associated with PC1 and words such as “disgusting”, “horrible”, “gross” and “unpleasant” confirming the strong negative valence. Meat, egg, and fried foods were closer to the origin, and the other less frequent triggers were at the negative extreme of PC1. Faecal odour (“poo”), however, is separated on PC2, associated with terms such as “weird”, “less unpleasant”, and “not bad” (and “better” and “pleasant” not shown), which have significant components on PC2.

**Figure 3:**
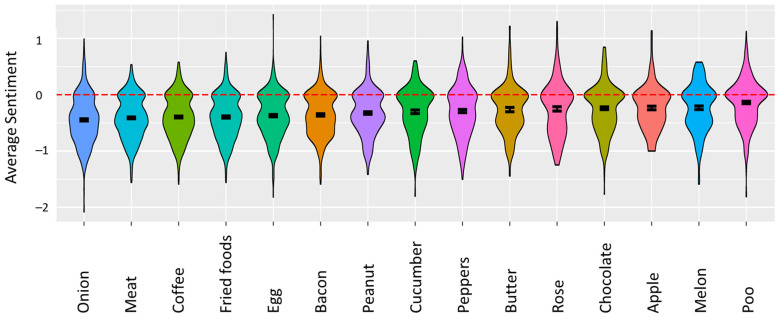
Sentiment analysis of the 14 set triggers and faecal odour. Distribution of sentiment for triggers is ordered from low to high sentiment. All triggers are below neutral sentiment (zero or red dashed line), with some (left side) being more negative than others (right side). Error bars represent standard errors.

This reverse change in valence has been discussed before [8,15], but here, we can show some objective evidence from a larger survey where we have directly asked respondents to rate the distortion and disgust of faecal odour alongside common food triggers. Sentiment analysis of the descriptors generated for the distortions is presented in Figure 3. Faecal odour ranks as more positive than any of the other 14 triggers, akin to the sentiments expressed for apple, melon, and chocolate, whereas onion, meat, and coffee rank as the most negative.

#### 3.3.6. Additional Triggers of Distortion (CATA)

The data from the CATA question showed that the 20 pre-selected items were each identified as triggers at least 80 times, so even those that had been selected as safe foods were reported as distorted in at least 10% of cases. These data, which were combined with the data from the 14 set triggers, and the frequency of distortions (Table 5), demonstrate that triggers are by no means universal, and therefore, our understanding of food consumption by those with parosmia cannot be considered straightforward. Unsurprisingly, garlic ranks high up the list with onion, but room freshener, cola drinks, and petrol are also high on the list, indicating it is not just the aroma molecules present in food (particularly cooked foods) that trigger the distortions. Although specific aroma compounds generated via the Maillard reaction during cooking are known to consistently trigger distortions in those suffering from parosmia [8], Table 5 suggests that there are other trigger molecules to be found. The bottom of the table is dominated by fresh fruit and nuts, indicating that for many, these may be safe foods.

#### 3.3.7. Additional Triggers (Free Text)

Analysis of the free choice answers reported well over 220 additional triggers, almost to the point that no food remained unmentioned. These were counted, and the 87 most frequently reported are listed in Table A1. What was clear from this section was the impact on people’s experience of non-food items, particularly those related to daily personal care activities such as showering and oral care. Water was mentioned as a trigger by 44 people and air by 14. For 41, who reported that everything was distorted, there was no respite from parosmia.

### 3.4. Analysis of the Free Text Question

Free text answers to the survey question “Do you have anything else you would like to tell us” gave us more detail of the impact of living with parosmia. Several themes reccurred, and a selection of poignant comments is quoted below. A number of comments related to the difficulty in describing the experience of parosmia were found in the free text:

*“It is really hard to find words to describe the new smells. I talked to my doctor three times since March, and his suggestions underlines the need for more knowledge and awareness. He answered me as if he really did not understand that the smells are different now …”.*

Several comments referred to “the COVID smell” or “Parosmia smell”. At onset, this seemed to be one single smell, and then with time, this experience diverged into two or more different “COVID smells” that could be roughly grouped together based on food type:

*“I only sense one unpleasant smell. Everything that has distorted smell, smells almost the same”.*

*“I seem to have two types of distortion ‘categories’—coffee, chocolate, onions etc taste like a musky, nutty, rancid, earthy taste. And things like peppers and melon taste more chemical, like something that would be flammable”.*

Consternation over the perceptions of disgust in the face of food was a common theme in the free text. This also extended to body odours, with a reversal of valence. What once smelled good (food) began to smell like body waste, and body waste became less offensive. This was hard for respondents to rationalise:

*“For me, my feaces [sic], urine and sweat have the same bad smell like the other distorted smells (like onion and fried meat). The bad smell is not familiar to me from before and it messes my head that my food smells like my body waste”.*

Burning nose and throat, and particularly nasty (metallic) tastes, were described in more detail:

*“Shampoos, cleaning products, soaps, perfumes, etc.,smell so strong I feel like my nose burns”.*

*“I have a weird constant taste in my mouth which matches the weird smell I keep smelling in everything”.*

A burning mouth has previously been associated with low body weight [27] and higher olfactory thresholds [28]. With aroma either absent or distorted, these additional sensations compound the problems associated with eating. With so many foods unbearably distorted, some commented on how their diet had changed:

*“My diet is quite limited at present so there may be other foods that smell distorted but I haven’t tried to eat them in the last week”.*

*“I am on a strict diet of fresh corn […], apricots, peaches, plums, grapes, cherries, cucumber, Fairlife protein shakes, diet Dr Pepper (coke and other sodas taste like pure dirt/mud), fresh mozzarella (all other hard cheeses, especially yellow cheddar, are rancid)”.*

As a result, those with long term parosmia may be subject to weight gain or weight loss depending on the severity of their symptoms and the range of foods that are tolerable:

*“Weight is increasing as the only tolerable foods are all sugary, stodgy, high fat, high calorie”.*

*“I am very sad and losing weight it feels worse every day that passes”.*

Fluctuating symptoms were one of the characteristics of post-COVID-19 olfactory dysfunction. Respondents to the free text question reported that fluctuations in symptoms caused considerable anxiety:

*“Distortion of smells fluctuates. Sometimes I can smell something and it smells normal, then I smell it again and it is distorted or I can’t smell it at all”.*

Another observation made by a number of respondents was that a “parosmic” smell, smelled once, might linger long after exposure to the item for hours or longer. This olfactory perseveration seems to suggest a malfunction of the attenuation system. It was not interrogated in the questionnaire but has been observed in social media threads for those with olfactory disorders [8].

*“The distorted odours fluctuate in strength but are never totally absent. Sometimes they linger even after the source is removed, and the memory of the odour alone can be enough to make me conscious of it, as though I can smell it, even if there is no odour source present”.*

Additionally, possibly associated with a malfunction of the attenuation system, “fleeting whiffs” were experienced. Unlike the “smell lock”, this was a quick flash of a perception that then disappeared, leaving people frustrated.

*“I still smell very little but sometimes have an initial weak or distorted ‘whiff’ of an item but on a second sniff I cannot smell anything. This has been happening since about 3 months after losing sense of smell”.*

Five respondents reported experiencing parosmia without associated anosmia or hyposmia.

*“I never lost my sense of smell. Parosmia came on suddenly. There was no absence of smell transition as implied in many questions”.*

Other studies have looked in detail at the emotional impact of parosmia [3,8,15]. The following quotes demonstrate the severity of the impact on quality of life:

*“Parosmia really affects mental health. Cannot eat out or socialise. … It is the weirdest thing but nobody believes you if you try to explain it. Cannot face the not knowing of how long it might last”.*

*“This needs to be over, it’s ruining my life and not worth getting up for this is severely effecting my way of life and it’s nice to know others care”.*

## 4. Discussion

The list of food ingredients that trigger distortions is long, almost to the point that no food is unmentioned. However, there are clear trends: The worst items are coffee, onion, and meat (worst being loosely defined as a combination of how frequently they were detected, how frequently they were distorted, how frequently they invoked disgust, and how intensely they were perceived). We know that coffee, roasted meat and fried foods have many molecules in common that trigger distortions and have similar formation pathways [10]. Certain pyrazines and certain sulphur-compounds which are formed in the Maillard reaction during the processing/cooking of food have been shown to elicit a “parosmia-like” smell. Since these Maillard reaction products are formed during roasting, frying, grilling, or baking of almost any food, those sensitive to coffee, meat and fried foods are likely to find many cooked foods unpalatable. Milder cooking processes may mitigate the distortions to a certain extent. Additionally, many recipes include onion and/or garlic which contain a different volatile profile to those above, producing thiols and disulphides that are also likely to trigger distortions. Coffee, onions, garlic, fried foods, eggs, and (in a non-vegetarian lifestyle) meat constitute a major part of a typical weekly diet, certainly in western cultures, and it is clear how distortions and sense of disgust in these key items could have a serious impact on diet and nutrition.

Changes in diet with olfactory dysfunction have been discussed by several authors [29,30,31,32] and discussed in detail by Chaaban et al. [33]. The relationship is complex depending on the nature, duration, and aetiology of the olfactory dysfunction [29] and can lead to the adoption of both healthier diets of better nutritional quality and diets high in sugar, fat, and salt. More recently, a paper addressing olfactory dysfunction as a result of COVID-19 showed a tendency for the diets to be higher in energy, fat, and sugar [34], but this is not necessarily the case for those with parosmia. The literature on the impact of parosmia on diet is scarce. Burges Watson et al. [3] reported a shift in appetite and intake in both directions: those with olfactory loss having a tendency for high energy diets and increased intake in search of the hedonic pleasure normally associated with food, but for those with parosmia, there was a tendency to avoid eating, leading to dramatic weight loss and further impacts on quality of life and mental health. The fact that key proteins such as meat (including bacon) and eggs are such strong triggers can result in a poor low-protein diet for those with parosmia unless suitable alternatives are sought. For some, safe foods are the less frequent triggers, such as fresh fruit and vegetables. However, safe foods vary from person to person and for some, diets consisting of relatively safe foods mean a diet of “plain potatoes, yogurt and cheese” or “bread, cheese, chips and cake” as reported in [8], resulting in weight gain. The added impact of a continuous metallic taste in the mouth, nose burn, and throat burn only exacerbates these problems.

However, triggers extend beyond food and the kitchen to homecare or personal care products and environmental odours, even water and air, contributing relentlessly to the misery experienced by those with parosmia, to such an extent that for some, there is no safe space either inside or outside of the home. The inability to describe the experience contributes to the frustration of having parosmia. Not only do patients suffer a daily onslaught of relentless and disagreeable smells, but they are not able to summon up enough descriptors to engage their doctors and support circles on the subject. In fact, a constant reminder of being unwell through triggers is a major reason individuals with parosmia may suffer more than those with a simple loss [15,35].

There is mounting literature in which the mechanisms by which SARS-CoV-2 may affect olfactory function are discussed [23,36,37], but few address the underlying mechanism of parosmia. Parosmia has sometimes been loosely characterised as a cross-wiring between the regenerating olfactory receptors and the glomeruli or olfactory bulb [23]. However, many have asked why these distortions are predominantly unpleasant, leading some to conjecture that the distortions of smell may be unpleasant because of a violation of expectations of how the odour of a familiar food or household item is usually perceived. However, this study shows that not all distorted and hence unexpected smells are found to be unpleasant. Thus, merely having an unexpected smell is not sufficient to cause disgust. Since the main triggers are experienced as having a disgusting odour, we need to account for what it is about the nature of the distorted odours that makes them disgusting. Analogies are made to the rotten, earthy, burnt, or chemical smells, but many participants in the study talk about a novel odour, or “that parosmia smell”. What is noticeable from the results is the high-intensity scores for those items found to be most disgusting; although this can fluctuate, the intensity and the disgust are correlated. Whatever way the participants are perceiving distorted odours, when they are found to be disgusting, they are perceived to be intense odours. The question remains as to whether their intensity is part of what makes them disgusting or whether it is because people find the novel or distorted odour to be disgusting that they also find it intense. Another hypothesis is that people with parosmia are becoming especially sensitive to particular molecules in a mixture, which means those compounds stand out and are therefore perceived as intense, or that the absence of other aromas that usually mask or round them out leaves the remaining compounds to smell more intense than they would in a mixture.

The reversal in valence described for faecal matter/body waste is disturbing. It has been explained by us previously [10] in terms of molecular triggers. Limited studies demonstrated that those with parosmia did not perceive the normally overpowering smell of indole, skatole, or cresol in faecal matter and therefore perceived only the aroma compounds which are normally masked—these could be pleasant aroma molecules or other trigger molecules.

It is also fascinating to note that some of the distorted items were still rated as pleasant despite the distortion: rose (50% of the time), apple (31%), and butter (29%). In cases where rose was rated as pleasant, it was rated as weaker in 50% of responses and only stronger in 6% of cases. In such cases, it may be that as a result of only partial regeneration of the olfactory sensory neurons, and consequently higher odour thresholds, most of the constituent aroma compounds are present at concentrations closer to the threshold than usual, with some dropping below the threshold, changing the balance and providing the distortion. This may be an instance of incomplete odour characterisation, as proposed by Leopold [35], where there is an imbalance in the aroma profile, which leads to the perceived distortion, but no single molecules trigger the switch in valence.

Rose may be an example of what can be termed “eusomia”, which has in the past been used to describe distortions which are pleasant, as opposed to cacosmia where olfactory distortions are negatively perceived [4]. Indeed, 56 of the 727 participants reported distorted items but no change in valence in any of them. Currently, the working definition of parosmia is generally inclusive of both eusomia and cacosmia, describing the twisting and warping of the sense of smell, without mention of the hedonic aspect [4], but there may be instances where the use of more precise terminology could be useful.

## 5. Conclusions

Here, we show that post-infectious olfactory dysfunction leads to significant distortion and hedonic change in key food items, predominantly but not exclusively towards a negative valence. Such intense distortions, the associated change in valence, loss of expected pleasure, and the presence of strange tastes and burning sensations certainly lead to changes in eating behaviours and serious longer-term consequences for mental health and quality of life. It remains to be seen whether there are any changes in the prevalence and trajectory of parosmia arising from the newer variants of COVID-19.

In subsequent work, we have looked in detail at the individual molecules which trigger distortions for a wide range of food [10], and we discuss in more detail how this can be rationalised with prevailing mechanistic theories. Further work is underway to test our hypothesis that some distortions, particularly those that do not elicit a sense of disgust, may be due to incomplete odour characterisation, as initially proposed by Leopold [35], whereas those that are associated with severe disgust are triggered by individual molecules. Future work should be targeted at understanding how individual molecules relay such aversions to the integrative centres in the brain.

## Figures and Tables

**Table 1:** Demographics and Aetiology of Parosmia.

Statistic		Count	Percent
Total Respondents		727	
Sex	Male	76	10%
	Female	651	90%
Age	Range (years)	18–75	
	Mean (years)	43	
Country of Residence	UK	330	45%
	USA	297	41%
	Canada	15	2%
	Spain	9	1%
	The Netherlands	8	1%
	Others (<1%)	68	9%
Ethnicity	White European	396	54%
	White North American	205	28%
	White Other	30	4%
	Other Ethnic Group	20	3%
	White South American	17	2%
	South Asian	11	2%
	Prefer not to say	10	1%
	Others (<1%)	38	5%
Smoking status	Smoker	52	7%
	Non-smoker	531	73%
	Ex-smoker	144	20%
Aetiology	COVID-19 (diagnosed)	367	50%
	COVID-19 (self-diagnosed)	239	33%
	Viral non-COVID-19 pre-Dec 2019	58	8%
	Viral non-COVID-19 post-Dec 2019	5	1%
	Accident, head/brain injury	14	2%
	Unexplained (idiopathic)	12	2%
	Other	7	1%
	Do not know	25	3%
Severity of COVID-19	Loss of smell only	66	11%
	Mild	194	32%
	Moderate	226	37%
	Severe	117	19%
	Very severe (hospitalised)	3	0.5%

**Table 2:** Timings of Smell Loss for All Post-Viral Cases.

	COVID-19 *n* = 606	Non-COVID-19 *n* = 63
very suddenly, before the other symptoms of infection appeared	127 (21%)	1 (2%)
very suddenly, during the infection	344 (57%)	29 (46%)
very suddenly, after the infection	69 (11%)	9 (14%)
gradually, I only noticed it was gone when I was recovering from the infection	66 (11%)	24 (28%)

**Table 3:** Intermittent Recovery of Olfactory Function.

	COVID-19	Non-COVID-19
no recovery of normal sense of smell	108 (18%)	46 (73%)
just a few hints that a sense of smell was returning and nothing else	171 (28%)	11 (17%)
partial recovery of a normal sense of smell	206 (34%)	5 (8%)
full recovery of a normal sense of smell	121 (20%)	1 (2%)

**Table 4:** Frequency of Food Items being Reported as (a) Distorted, (b) Not Detected, or (c) Normal.

Item	Reported as Distorted Freq (%) ^1^	Item	Reported as Not Detected Freq (%) ^1^	Item	Reported as Normal Freq (%) ^1^
Butter	18	Coffee	7	Meat	11
Apple	23	Onion	18	Coffee	11
Rose	25	Meat	18	Onion	12
Cucumber	29	Fried foods	18	Egg	18
Melon	32	Bacon	21	Fried foods	20
Peppers	36	Peanuts	24	Bacon	22
Chocolate	43	Chocolate	27	Peanuts	26
Peanuts	50	Egg	29	Chocolate	30
Egg	53	Cucumber	30	Peppers	31
Bacon	57	Peppers	33	Melon	34
Fried foods	61	Melon	33	Rose	36
Onion	70	Apple	36	Butter	39
Meat	71	Rose	39	Cucumber	40
Coffee	82	Butter	44	Apple	41

^1^ Percentage frequency excluding those who are not familiar with or do not consume the item.

**Table 5:** Frequency of Items being Reported as Distorted (20 from the CATA question with the 14 preset triggers in italics).

Distorted Aromas	Count ^1^	Distorted Aromas (Contd)	Count ^1^	Distorted Aromas (Contd)	Count ^1^
*Coffee*	570	*Peanuts*	301	Celery	142
Garlic	496	Bananas	289	Carrots	131
*Onion*	495	Popcorn	288	Peaches	130
*Meat*	490	Cigarettes	277	Vanilla	130
Room freshener	435	*Peppers*	217	Mango	122
*Fried foods*	424	Tomatoes	204	Hazelnuts	122
Cola drinks	356	*Cucumber*	178	*Butter*	120
*Egg*	350	*Melon*	171	Walnuts	115
*Bacon*	340	*Rose*	148	Grapefruit	112
Toast	336	*Apple*	147	Passionfruit	84
Petrol	318	Raspberries	146	Honey	80
*Chocolate*	302				

^1^ Count = number of times scored as distorted.

## Data Availability

The data presented in this study are openly available in the University of Reading Research Data Archive at http://doi.org/10.17864/1947.000367, last accessed on 15 February 2022.

## Supplementary Materials

The following supporting information can be downloaded at: https://www.mdpi.com/article/10.3390/foods11070967/s1, Table S1: Descriptors for 14 Set Triggers.

## Appendix A

**Table A1:** List of triggers found in personal care and homecare products, environment, foods, and beverages.

Other Distorted Smells Personal Care	Count ^1^	Other Distorted Smells Home and Environment	Count ^1^	Other Distorted Smells Foods and Beverages	Count ^1^
***Daily washing/*** ***grooming (445)***		***Home cleaning (223)***		***Herbs (119)***	
soap	132	cleaning products	110	mint	81
deodorants/antiperspirant	106	detergents	75	herbs	15
shower gel	91	fabric softner	38	basil	12
personal care products	63	***Sanitisers (98)***		rosemary	11
cosmetics and toiletries	35	sanitisers	46	***Fruit (114)***	
nail care	12	bleach	39	citrus (lemon, orange, lime)	83
sun cream	6	wipes	13	strawberries	31
***Personal Fragrances (272)***		***Environment (96)***		***Alcoholic beverages (103)***	
perfume	223	water	44	wine	63
candles	30	air	14	beer	22
aftershave	19	garbage	10	rubbing alcohol	8
***Hair care (247)***		chlorine	9	gin	7
shampoo	178	coast	8	cider	3
conditioner	44	rain/petrichor	6	***Carbohydrates (101)***	
hair products	25	manure	4	bread	46
***Oral care (146)***		tarmac	1	cookies biscuits	15
toothpaste	133	***Essential oils (95)***		baked goods	14
mouthwash	13	other essential oils	39	rice, rice cakes	12
***Body aromas (132)***		eucalyptus/Vicks	27	pasta	11
other body odour	76	lavender	27	tortilla	3
urine	44	rose essential oil	2	***Dairy (55)***	
breath	7	***Garden (90)***		cheese	24
new born baby/husband	5	grass	47	tea	15
		flowers	20	milk	11
		plants, leaves	12	dairy	5
		soil, earth	6	***Vegetables and pulses (54)***	
		trees	5	brassica	21
		***Miscellaneous (58)***		salad	15
		everything	41	peas and beans, pinto	14
		marijuana	17	chickpeas	2
		***Car (43)***		soya	2
		petrol/diesel fumes	42	***Spices (48)***	
		plastic/interior	1	coriander, cilantro	19
		***Pets (32)***		ginger (beer, tea, lotion)	9
		petfood	16	mustard	5
		pet	13	clove	4
		cat litter	3	cinnamon	4
		***Smoke (28)***		cumin	4
		smoke	22	paprika	3
		bonfire	6	***Fish (18)***	
		***Home (4)***		fish, tuna	18
		pens/crayons	2		

^1^ Count = number of times scored as distorted.

## References

[1] Spence C. (2015). Just how much of what we taste derives from the sense of smell?. Flavour.

[2] Croy I., Nordin S., Hummel T. (2014). Olfactory Disorders and Quality of Life—An Updated Review. Chem. Senses.

[3] Burges Watson D.L., Campbell M., Hopkins C., Smith B., Kelly C., Deary V. (2020). Altered Smell and Taste: Anosmia, parosmia and the impact of long COVID-19. PLoS ONE.

[4] Hummel T., Whitcroft K.L., Andrews P., Altundags A., Cinghi C., Costanzo R.M., Damm M., Frasnelli J., Gudziol H., Gupta N. (2017). Position paper on olfactory dysfunction. Rhinol. Suppl..

[5] Keller A., Malaspina D. (2013). Hidden consequences of olfactory dysfunction: A patient report series. BMC Ear Nose Throat Disord..

[6] Hopkins C., Surda P., Vaira L.A., Lechien J.R., Safarian M., Saussez S., Kumar N. (2020). Six month follow-up of self-reported loss of smell during the COVID-19 pandemic. Rhinology.

[7] Olofsson J.K., Ekesten F., Nordin S. (2021). Smell distortions: Prevalence, longevity and impact of parosmia in a population-based, longitudinal study spanning 10 years. PsyArXiv.

[8] Parker J.K., Kelly C.E., Kirkwood A.F., Smith B.C., Hopkins C., Gane S. (2021). Patients’ Perspectives on Qualitative Olfactory Dysfunction: Thematic Analysis of Social Media Posts. JMIR Form. Res..

[9] Teaima A.A., Salem O.M., Teama M., Mansour O.I., Taha M.S., Badr F.M., Khater S.S., Abdou K., Mahmoud M.S. (2022). Patterns and clinical outcomes of olfactory and gustatory disorders in six months: Prospective study of 1031 COVID-19 patients. Am. J. Otolaryngol..

[10] Parker J.K., Kelly C.E., Gane S.B. (2021). Molecular Mechanism of Parosmia. medRxiv.

[11] Ooms J. (2020). Hunspell: High-Performance Stemmer, Tokenizer, and Spell Checker (3.0.1) [Computer Software]. https://cran.r-project.org/web/packages/hunspell/index.html.

[12] Pennec E., Slowikowski K. (2018). Ggwordcloud: A Word Cloud Geom for ‘ggplot2’, 0.3.0 [Computer Software]. https://cran.r-project.org/web/packages/ggwordcloud/vignettes/ggwordcloud.html.

[13] Rinker T.W. (2019). Sentimentr: Calculate Text Polarity Sentiment, (2.7.1) [R Package]. https://github.com/trinker/sentimentr.

[14] Koyama S., Ueha R., Kondo K. (2021). Loss of Smell and Taste in Patients with Suspected COVID-19: Analyses of Patients’ Reports on Social Media. J. Med. Internet Res..

[15] Pellegrino R., Mainland J.D., Kelly C.E., Parker J.K., Hummel T. (2021). Prevalence and Correlates of Parosmia and Phantosmia among Smell Disorders. Chem. Senses.

[16] Hong S.-C., Holbrook E.H., Leopold D.A., Hummel T. (2012). Distorted olfactory perception: A systematic review. Acta Oto-Laryngol..

[17] Vaira L.A., Hopkins C., Sandison A., Manca A., Machouchas N., Turilli D., Lechien J.R., Barillari M.R., Salzano G., Cossu A. (2020). Olfactory epithelium histopathological findings in long-term coronavirus disease 2019 related anosmia. J. Laryngol. Otol..

[18] Borsetto D., Hopkins C., Philips V., Obholzer R., Tirelli G., Polesel J., Boscolo-Rizzo P. (2020). Self-reported alteration of sense of smell or taste in patients with COVID-19: A systematic review and meta-analysis on 3563 patients. Rhinology.

[19] Gane S.B., Kelly C., Hopkins C. (2020). Isolated sudden onset anosmia in COVID-19 infection. A novel syndrome?. Rhinology.

[20] Lechien J.R., Chiesa-Estomba C.M., Beckers E., Mustin V., Ducarme M., Journe F., Marchant A., Jouffe L., Barillari M.R., Cammaroto G. (2021). Prevalence and 6-month recovery of olfactory dysfunction: A multicentre study of 1363 COVID-19 patients. J. Intern. Med..

[21] Lechien J.R., Chiesa-Estomba C.M., De Siati D.R., Horoi M., Le Bon S.D., Rodriguez A., Dequanter D., Blecic S., El Afia F., Distinguin L. (2020). Olfactory and gustatory dysfunctions as a clinical presentation of mild-to-moderate forms of the coronavirus disease (COVID-19): A multicenter European study. Eur. Arch. Oto-Rhino-L.

[22] Yan C.H., Faraji F., Prajapati D.P., Ostrander B.T., DeConde A.S. (2020). Self-reported olfactory loss associates with outpatient clinical course in COVID-19. Int. Forum Allergy Rhinol..

[23] Cooper K.W., Brann D.H., Farruggia M.C., Bhutani S., Pellegrino R., Tsukahara T., Weinreb C., Joseph P.V., Larson E.D., Parma V. (2020). COVID-19 and the Chemical Senses: Supporting Players Take Center Stage. Neuron.

[24] Parma V., Ohla K., Veldhuizen M.G., Niv M.Y., Kelly C.E., Bakke A.J., Cooper K.W., Bouysset C., Pirastu N., Dibattista M. (2020). More than smell—COVID-19 is associated with severe impairment of smell, taste, and chemesthesis. Chem. Senses.

[25] Alon E.E., Glikson E., Shoshani Y., Dobriyan A., Yahalom R., Yakirevitch A. (2021). Six-month smell and taste recovery rates in coronavirus disease 2019 patients: A prospective psychophysical study. J. Laryngol. Oto..

[26] Ohla K., Veldhuizen M.G., Green T., Hannum M.E., Bakke A.J., Moein S.T., Tognetti A., Postma E.M., Pellegrino R., Hwang L.-D. (2021). Increasing incidence of parosmia and phantosmia in patients recovering from COVID-19 smell loss. Rhinology.

[27] Deems D.A., Doty R.L., Settle R.G., Moore-Gillon V., Shaman P., Mester A.F., Kimmelman C.P., Brightman V.J., Snow J.B. (1991). Smell and Taste Disorders, A Study of 750 Patients from the University of Pennsylvania Smell and Taste Center. Arch. Otorhinolaryngol. Head Neck Surg..

[28] Siviero M., Teixeira M.J., Siqueira J.T., Siqueira S.R. (2011). Central mechanisms in burning mouth syndrome involving the olfactory nerve: A preliminary study. Clinics.

[29] Stevenson R.J., Mahmut M.K., Horstmann A., Hummel T. (2020). The Aetiology of Olfactory Dysfunction and Its Relationship to Diet Quality. Brain Sci..

[30] Zang Y., Han P., Burghardt S., Knaapila A., Schriever V., Hummel T. (2019). Influence of olfactory dysfunction on the perception of food. Eur. Arch. Oto-Rhino-L.

[31] Kershaw J.C., Mattes R.D. (2018). Nutrition and taste and smell dysfunction. World J. Otorhinolaryngol. Head Neck Surg..

[32] Pellegrino R., Hummel T., Emrich R., Chandra R., Turner J., Trone T., Dorminy C., Luckett C.R. (2020). Cultural determinants of food attitudes in anosmic patients. Appetite.

[33] Chaaban N., Hoier A.T.Z.B., Andersen B.V. (2021). A Detailed Characterisation of Appetite, Sensory Perceptional, and Eating-Behavioural Effects of COVID-19: Self-Reports from the Acute and Post-Acute Phase of Disease. Foods.

[34] Rawal S., Duffy V.B., Berube L., Hayes J.E., Kant A.K., Li C.-M., Graubard B.I., Hoffman H.J. (2021). Self-Reported Olfactory Dysfunction and Diet Quality: Findings from the 2011-2014 National Health and Nutrition Examination Survey (NHANES). Nutrients.

[35] Leopold D. (2002). Distortion of olfactory perception: Diagnosis and treatment. Chem. Senses.

[36] Doty R.L. (2021). The mechanisms of smell loss after SARS-CoV-2 infection. Lancet Neurol..

[37] Brann D.H., Tsukahara T., Weinreb C., Lipovsek M., Van den Berge K., Gong B., Chance R., Macaulay I.C., Chou H.J., Fletcher R.B. (2020). Non-neuronal expression of SARS-CoV-2 entry genes in the olfactory system suggests mechanisms underlying COVID-19-associated anosmia. Sci. Adv..

